# Meningitis in adult patients with a negative direct cerebrospinal fluid examination: value of cytochemical markers for differential diagnosis

**DOI:** 10.1186/cc10254

**Published:** 2011-06-06

**Authors:** Alain Viallon, Nicolas Desseigne, Olivier Marjollet, Albert Birynczyk, Mathieu Belin, Stephane Guyomarch, Jacques Borg, Bruno Pozetto, Jean Claude Bertrand, Fabrice Zeni

**Affiliations:** 1 Emergency and Intensive Care Units, North Hospital, Centre Hospitalier Universitaire de Saint-Etienne, Saint-Etienne, 42055, Cedex 2, France; 2 Biochemical Laboratory, North Hospital, Centre Hospitalier Universitaire de Saint-Etienne, Saint-Etienne, 42055, Cedex 2, France; 3 Microbiology Laboratory, North Hospital, Centre Hospitalier Universitaire de Saint-Etienne, Saint-Etienne, 42055, Cedex 2, France

## Abstract

**Introduction:**

The objective of this study was to determine the ability of various parameters commonly used for the diagnosis of acute meningitis to differentiate between bacterial and viral meningitis, in adult patients with a negative direct cerebrospinal fluid (CSF) examination.

**Methods:**

This was a prospective study, started in 1997, including all patients admitted to the emergency unit with acute meningitis and a negative direct CSF examination. Serum and CSF samples were taken immediately on admission. The patients were divided into two groups according to the type of meningitis: bacterial (BM; group I) or viral (VM; group II). The CSF parameters investigated were cytology, protein, glucose, and lactate; the serum parameters evaluated were C-reactive protein and procalcitonin. CSF/serum glucose and lactate ratios were also assessed.

**Results:**

Of the 254 patients with meningitis with a negative direct CSF examination, 35 had BM and 181, VM. The most highly discriminative parameters for the differential diagnosis of BM proved to be CSF lactate, with a sensitivity of 94%, a specificity of 92%, a negative predictive value of 99%, a positive predictive value of 82% at a diagnostic cut-off level of 3.8 mmol/L (area under the curve (AUC), 0.96; 95% confidence interval (CI), 0.95 to 1), and serum procalcitonin, with a sensitivity of 95%, a specificity of 100%, a negative predictive value of 100%, and a positive predictive value of 97% at a diagnostic cut-off level of 0.28 ng/ml (AUC, 0.99; 95% CI, 0.99 to 1).

**Conclusions:**

Serum procalcitonin and CSF lactate concentrations appear to be the most highly discriminative parameters for the differential diagnosis of BM and VM.

## Introduction

The emergency therapeutic management of meningitis involves rapid identification of the bacterial or viral nature of this disease, so that antibiotic treatment can be started without delay [1], even though the relation between prognosis and delay in starting antibiotic therapy has not been clearly established [2-5].

The immediate identification of bacterial meningitis (BM) is based on direct examination of the cerebrospinal fluid (CSF) or the detection of bacterial antigens in the CSF. These tests have a low sensitivity, and in 30% to 50% of cases, they do not contribute to differential diagnosis [6-9].

A model for predicting the bacterial origin of meningitis was proposed by Hoen *et al*., [10-13], but the results of external validation tests were not homogeneous.

The preliminary results of our study suggested that serum procalcitonin and CSF lactate levels were better markers than were those classically used with regard to differentiating between BM and viral meningitis (VM) in patients with meningitis and a negative direct CSF examination [13]. The objective of this new analysis was to test the validity of these preliminary results.

## Materials and methods

This was a prospective study, including all adult patients admitted to the emergency unit with suspected meningitis from January 1997 onward. Patients included in this study provided written informed consent. However, and in accord with the French law, this study did not required ethical approval because of the observational nature of study. This study received the agreement of the French Data Protection Authority.

Lumbar puncture was performed immediately on the patient's admission to the emergency unit. Patients with meningitis, defined by a leukocyte count > 5 per mm^3 ^in the CSF, were eligible for inclusion in the study. Exclusion criteria comprised the presence of bacteria in the CSF evidenced by direct examination and/or detection of bacterial antigens in the CSF, antibiotic treatment before admission (more than two successive doses of the prescribed antibiotic), the presence of another focus of infection in addition to meningitis, and meningitis finally assumed to be of bacterial origin, despite the absence of microbiologic documentation, and treated with antibiotics during the patient's hospitalization.

Blood tests (complete blood count, C-reactive protein (CRP), lactate, procalcitonin (PCT), electrolytes, blood cultures), and CSF analyses (cytology, bacteriology, lactate, protein, glucose) were performed on the admission of the patient, before the start of any antibiotic treatment. The limits of detection were 0.07 ng/ml for PCT (Kriptor; Brahms, France), 4 mg/L for CRP (turbidimetric method; Diagam, France) and 1.25 mmol/L for lactate (i-STAT; Abbott, USA). The CSF/serum glucose and lactate ratios also were calculated. Between 1997 and 1999, neurotropic viruses were identified on the basis of two serologic examinations separated by an interval of 15 days and/or detection of the viral genome by using a polymerase chain reaction (PCR) amplification technique. After 1999, only PCR tests were used for virus identification in the CSF.

BM was diagnosed on the basis of a positive bacterial culture of CSF. The diagnosis of viral meningitis or meningoencephalitis was confirmed in the absence of any bacteria detectable by direct CSF examination or in bacterial cultures, and a positive viral serology or PCR test; in the absence of proven viral etiology, meningitis was considered to be viral if a cure was achieved without any antibiotic treatment apart from antiviral therapy. In view of their practically identical cytologic and chemical characteristics, VM and viral meningoencephalitis (VME) were combined in a single VM group. The confirmed diagnosis was that recorded on the patient's discharge from hospital. From 1997 onward, all patients with meningitis were contacted between 28 and 30 days after their discharge from hospital to obtain information on the following items: need for readmission to hospital, new antibiotic prescription, and persistence or recurrence of headache.

### Statistical analysis

To determine the value of the different parameters for the differential diagnosis of BM and VM, the patients were divided into two groups: those with BM (group I) and those with VM (group II). The results were expressed as the mean ± standard deviation. Mean values were compared by using the Mann-Whitney nonparametric test, the threshold of statistical significance being set at *P *< 0.05. The discriminative power of the various parameters studied was determined by means of receiver operating characteristic (ROC) curves. The diagnostic cut-off level for differentiation of BM and VM was defined according to the maximum value of the Youden index. The ROC curves were compared by using Hanley's method [14].

## Results

Between January 1997 and December 2009, 97 patients with BM and 218 patients with VM (183 with VM and 35 with VME) were admitted to the emergency unit of the Saint-Etienne University Hospital.

Among the 97 patients with BM, 62 cases were excluded: 42 had a positive direct CSF examination, 16 had an additional focus of infection (pulmonary (eight), in the ear, nose, and throat region (three), ascitic (two), intra-abdominal (two), one had a spondylitis with discitis), and four patients had meningitis assumed to be of bacterial origin in the absence of microbiologic documentation. In the CSF samples taken from these four patients, the polynuclear neutrophil count ranged from 964 to 1,480/mm^3^; protein, from 3.8 to 8.3 g/L; glucose, from 1 to 2.8 mmol/L; and lactate, from 5 to 9.2 mmol/L. These four patients received antibiotic treatment for 18 to 22 days and were cured with no sequelae. A CSF fistula was diagnosed in one of the four patients.

Thirty-five patients with BM and a negative direct CSF examination were included in this study. The following bacteria were identified in CSF cultures: *Streptococcus pneumoniae *(14), *Listeria monocytogenes *(6), *Neisseria meningitidis *(5), *Streptococcus *sp. (4), *Haemophilus influenzae *(2), *Staphylococcus aureus *(2), and other species (2). A single bacterial species was identified in the blood cultures of 14 patients.

Among the 218 patients with VM, the virus was identified in 85 patients (39%): enterovirus (53), herpes virus (25), varicella zoster virus (five), and *Paramyxovirus *(2). In eight of these patients, admitted prior to 1999, virus identification was based on serology alone.

The main clinical characteristics of the patients are presented in Table 1.

**Table 1:** Clinical characteristics of patients on admission expressed as means (± standard deviation) or numbers (%)

Characteristics	Bacterial meningitis *n *= 35	Viral meningitis *n *= 218	*P*
Demographic characteristics			
Mean age (years)	55 ± 20	35 ± 18	ns
Male, *n *(%)	17 (48)	116 (53)	ns
Clinical characteristics			
Temperature, *n *(%)	38.9 ± 0.2	39.1 ± 0.2	ns
Headache, *n *(%)	14 (40)	158 (72)	0.0001
Nuchal rigidity, *n *(%)	16 (46)	121 (55)	ns
Seizures, *n *(%)	3 (9)	4 (2)	0.02
Focal neurologic signs, *n *(%)	2 (6)	6 (3)	ns
Confusion	11 (31)	31 (14)	0.01
CGS (mean score ± SD)	13 ± 2	14 ± 2	ns
SAPS II	18 ± 9	8 ± 4	0.0001

CGS, Coma Glasgow Scale; SAPS II, simplified acute physiologic score II.

The mean values of the serum parameters studied are shown in Table 2 for each group. With the exception of serum glucose, these values differed to a statistically significant extent between the two groups. CRP levels < 30 mg/L were noted in 10 patients in the BM group. CRP levels > 30 mg/L were seen in 25% of patients in the VM group. Only PCT values showed no overlap between the BM and VM groups. However, two patients with BM (bacterial species identified: *Listeria monocytogenes and Haemophilus influenzae*, respectively) had low PCT values: 0.20 and 0.28 ng/ml. The CSF samples taken from these two patients showed a neutrophil count < 100/mm^3 ^and normal glucose levels (> 3 mmol/L), but the other CSF parameters studied allowed confirmation of the bacterial origin of meningitis according to the diagnostic cut-off levels specified in this article. In all the other patients with BM, PCT levels were > 0.93 ng/ml.

**Table 2:** Mean values (± standard deviation) of biochemical serum parameters

	Bacterial meningitis *n *= 35	Viral meningitis *n *= 218	*P*
Glucose level (mmol/L) (min - max)	8.4 ± 2.7 (4.9-14.6)	6.1 ± 1.8 (4-18)	0.03
Lactate (mmol/L) (min - max)	3 ± 1.8 (1-8)	1.8 ± 1.7 (1-7)	0.0001
Procalcitonin (ng/ml) (min - max)	17 ± 45 (0.2-257)	0.09 ± 0.03 (0.07-0.1)	0.0001
C-reactive protein (mg/L) (min - max)	159 ± 148 (19-660)	42 ± 39 (3-152)	0.0001

The mean values of the CSF parameters studied and the CSF/serum glucose and lactate levels are presented in Table 3. With regard to CSF parameters, 50% of the patients in the BM group had a neutrophil count < 440/mm^3^, whereas > 10% of the patients in the VM group had a neutrophil count > 500/mm^3^. CSF glucose levels were not depressed in 25% of the patients with BM, but lower-than-normal CSF glucose levels were seen in 5% of patients with VM. CSF protein levels were elevated in > 25% of patients with VM and were normal in 5% of patients with BM.

**Table 3:** Mean values (± standard deviation) of cytologic and biochemical cerebrospinal fluid parameters

	Bacterial meningitis *n *= 35	Viral meningitis *n *= 218	*P*
Leucocyte count/mm^3^ (min - max)	1,515 ± 2,000 (25-10,320)	257 ± 520 (23-6,500)	0.0001
Neutrophil count/mm^3^ (min - max)	1,003 ± 2,000 (22-10,000)	75 ± 160 (15-1,188)	0.0001
Glucose level (mmol/L) (min - max)	2.5 ± 1.5 (0.1-5)	3.4 ± 1 (2.1-14)	0.0001
CSF/serum glucose ratio (min - max)	0.24 ± 0.3 (0.1-0.5)	0.51 ± 0.15 (0.3-1.8)	0.01
Lactate (mmol/L) (min - max)	9 ± 5 (3.2-25)	2.6 ± 1.6 (0.5-3.7)	0.0001
CSF/serum lactate ratio (min - max)	3.7 ± 2.7 (1.5-8.4)	1.5 ± 1.4 (1-1.9)	0.0001
Protein level (g/L) (min - max)	4.9 ± 4.6 0.5-24	1 ± 0.6 0.3-4	0.0001

**Figure 1:**
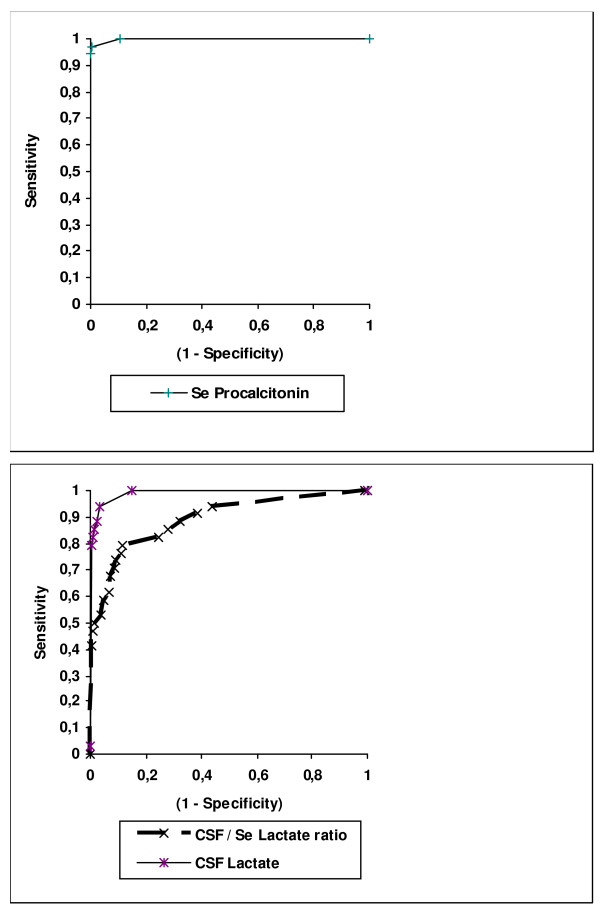
**Receiver operating characteristic (ROC) curves of the two most highly discriminative parameters (CSF, cerebrospinal fluid; Se, serum)**.

**Figure 2:**
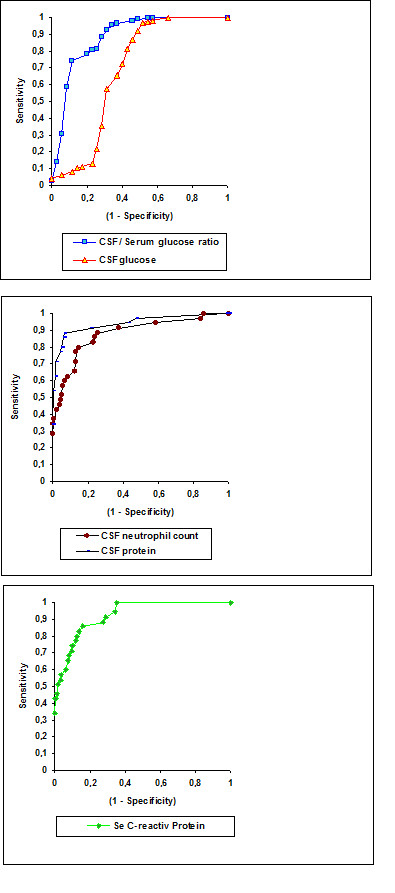
**Receiver operating characteristic (ROC) curves of the other parameters studied (CSF, cerebrospinal fluid; Se, serum)**.

The sensitivity, specificity, and positive and negative predictive values of the various parameters studied are presented in Table 4. The ROC curves are illustrated in Figures 1 and 2. Comparison of the ROC curves showed no statistically significant difference between PCT and CSF lactate (*P *= 0.1). In contrast, statistically significant differences were seen between PCT and CRP (*P *= 0.018), between PCT and CSF protein (*P *= 0.02), and between PCT and the other parameters.

**Table 4:** Diagnostic value of the various parameters studied

	CSF parameters				Serum parameters		CSF/serum ratio	
	**Neutrophil/mm^3^**	**Protein g/L**	**Glucose mmol/L**	**Lactate mmol/L**	**PCT ng/ml**	**CRP mg/l**	**Glucose CSF/serum**	**Lactate CSF/serum**

AUC	0.86	0.93	0.69	0.96	0.99	0.92	0.87	0.84
[CI 95%]	[0.86-0.94]	[0.92-0,98]	[0.69-0.76]	[0.95-1]	[0.99-1]	[0.92-0.98]	[0.86-0.91]	[0.84-0.93]
Optimal cut-point	118	1.88	2.2	3.8	0.28	37	0.48	2.22
Sensitivity	0.80	0.89	0.97	0.94	0.97	0.86	0.84	0.79
Specificity	0.85	0.93	0.49	0.97	1	0.84	0.89	0.89
Positive predictive value	0.47	0.67	0.92	0.82	0.97	0.46	0.98	0.52
Negative predictive value	0.96	0.98	0.71	0.99	1	0.97	0.66	0.97
Global value	0.84	0.92	0.90	0.96	0.99	0.84	0.76	0.87

AUC, area under a curve; CI, confidence interval; CSF, cerebrospinal fluid.

Finally, among the 183 patients with VM, 31 (17%) received antibiotic treatment for a period ranging from 8 to 24 hours on the assumption that their meningitis was of bacterial origin. All patients with BM received empirical antibiotic treatment in the emergency unit.

## Discussion

These results confirm the value of serum PCT and CSF lactate assays for the differentiation of bacterial and viral meningitis in adult patients with a negative direct CSF examination in the emergency unit.

Clinical signs have been shown to have a low sensitivity and specificity for the diagnosis of meningitis and are of no value for differentiating between meningitis of bacterial and viral origins [15]. In the absence of any contraindication, a lumbar puncture should always be performed in patients with suspected meningitis on clinical grounds [16]. However, in the emergency context, direct CSF examination provides evidence of bacterial meningitis in only 60% to 80% of cases [8,10]. Diagnosis is therefore based on the analysis of cytochemical CSF and serum parameters.

Few published studies have focused on the value of these parameters for the differential diagnosis of BM in patients with a negative direct CSF examination, so it is difficult to compare their results.

Spanos [8] described a study in 55 patients with BM and a negative direct CSF examination. The median neutrophil count in the CSF of these patients was 512/mm^3^, compared with 1,520/mm^3 ^in the CSF of 134 patients with BM and a positive direct CSF examination; the difference between these groups was statistically significant. Ray *et al*. [17] reported a mean neutrophil count of 428/mm^3 ^in 18 patients with BM and a negative direct CSF examination. In this study, neutrophil count had a sensitivity of 78% and a specificity of 75% for the differential diagnosis of BM. In our study, this parameter was among those most poorly discriminating between BM and VM, with a sensitivity of 80% and a specificity of 85% at a diagnostic cut-off level of 118/mm^3^.

Low CSF glucose levels (2 to 2.5 mmol/L) and a low CSF/serum glucose ratio (on the order of 0.30 to 0.40) have been classically described as a feature of BM [18-22]. Spanos [8] reported a median CSF glucose level of 3.4 mmol/L (CSF/serum glucose ratio, 0.45) in patients with BM and a negative direct examination of the CSF, compared with 1.7 mmol/L (CSF/serum glucose ratio, 0.23) in 134 patients with BM and a positive direct CSF examination. Similar values were seen in the patients included in our study, although CSF glucose levels were more markedly reduced.

Elevated CSF protein levels are also seen in patients with BM, mean values ranging from 1 to 2.5 g/L [8,17,21,23]. With regard to the differential diagnosis of BM, the reported sensitivity of this parameter ranged from 63% to 86% with a specificity of 94% to 98% [17,21]. Normal CSF protein values were found in 10% of patients with BM and a negative direct CSF examination [8]. In our study, a CSF protein level of 1.88 g/L gave a sensitivity of 89% and a specificity of 93% for the diagnosis of BM. Normal levels of protein in the CSF were seen in 5% of the patients with BM in our study.

Although several studies have demonstrated the value of determining lactate levels in the CSF for the differential diagnosis of BM, none of these focused specifically on the diagnostic value of this parameter in adult patients with BM and a negative direct CSF examination [21,23-27]. Furthermore, its use in this context was not recommended by the last-but-one French consensus conference on the management of BM, notably on the grounds of the disparity of the diagnostic cut-off levels reported and the methods used to determine these [28]. In addition, the selection of the control groups compared with the BM group could be a confounding factor with regard to interpretation of the results. In some studies, the BM group was compared with heterogeneous groups of patients with pathologic conditions likely to induce elevated lactate concentrations in the CSF (for example, brain tumor, subdural hemorrhage, trauma, and coma of metabolic origin) [19,27]. In studies comparing a group of patients with BM with a group of patients with VM, the cut-off level for the differential diagnosis of BM (correctly defined by ROC curves) ranged from 3.2 to 4 mmol/L with a sensitivity of 78% to 100% and a specificity of 96% to 100% [29-31]. In our study, with a diagnostic cut-off value of 3.8 mmol/L, the sensitivity for the differential diagnosis of BM was 94%, with a specificity of 97%. This was one of the two parameters that discriminated best between BM and VM in our study, based on comparisons between the different ROC curves. The most recent French consensus conference on the management of BM emphasizes the value of this marker [32].

The value of serum levels of C-reactive protein, a protein characteristic of the acute phase of inflammation, in differentiating BM and VM, was investigated in several published studies, as well as in a meta-analysis (including 14 studies in which serum levels of C-reactive protein were measured) [17,26,33-35]. The cut-off levels for the differential diagnosis of BM ranged from 20 to 100 mg/L [35]. The best compromise between sensitivity and specificity seemed to be achieved at diagnostic cut-off levels between 20 and 50 mg/L, although even in this case, the sensitivity and specificity were still less than 80%, notably with regard to adult patients with acute meningitis and a negative direct CSF examination [17]. In our study, the contribution of serum C-reactive protein to the diagnosis of BM was inferior to that of CSF protein and lactate and serum PCT, with a sensitivity of 86% and a specificity of 84% at a diagnostic cut-off level of 37 mg/L.

PCT is another marker that has been evaluated with regard to its usefulness in distinguishing between infections of bacterial and viral origin. Serum levels of this marker increase within 2 hours after stimulation of inflammatory processes, as shown in healthy volunteers receiving an injection of lipopolysaccharides [36]. The first studies on PCT in the context of BM, in adults and children, were published in 1997 and 2000 [9,31,37,38]. Serum PCT was found to be capable of differentiating BM (23 cases in children and 32 in adults) from VM in 100% of cases, but the diagnostic cut-off levels used ranged from 0.20 ng/ml to 2 ng/ml. Other studies subsequently completed these data in adults with conflicting results. In the study reported by Schwarz *et al*. [39], among 16 patients with BM, five showed serum PCT levels < 0.5 ng/ml, the bacterial species identified in these patients being *Mycobacterium tuberculosis *(one), *Borrelia burgdorferi *(one), *Staphylococcus aureus *(one), *Haemophilus influenzae *(one), and *Streptococcus pneumoniae *(one). The sensitivity of this parameter for the differential diagnosis of BM was 69%, and the specificity was 100% at a diagnostic cut-off level of 0.5 ng/ml. In another study on 12 patients with BM, reported by Hoffmann *et al*. [40], PCT was undetectable in the serum of three patients; in this study, the patients had postoperative meningitis rather than community-acquired BM. According to Jereb *et al*. [41], on the basis of 20 patients with BM, a diagnostic cut-off level of 0.5 ng/ml had a sensitivity of 90% and a specificity of 100% for the differential diagnosis of BM. In this study, PCT levels < 0.5 ng/ml were found in two patients with BM caused by *Listeria monocytogenes*. More recently, Ray *et al*. [17] published the results of a study in which PCT was assayed in serum from eight patients with BM and a negative direct CSF examination and 55 patients with VM. A diagnostic cut-off level of 2.13 ng/ml had a sensitivity of 87% and a specificity of 100% for the differential diagnosis of BM. The diagnostic cut-off value defined in our study was 0.28 ng/ml, giving a sensitivity of 97% and a specificity of 100%. However, this level was close to the threshold of detection of PCT, 0.07 ng/ml.

### Limitations of the study

The population selected for this study may be criticized. All patients with meningitis and a direct examination of CSF were included. We could have excluded patients whose CSF appeared turbid macroscopically, but this criterion may be subjective.

The causative virus was identified in only 39% of the patients with VM. Improvements in PCR techniques should make it possible to increase this rate in the future. Among the patients classified as having VM in our study, none received a prescription for an antibiotic within 30 days after discharge from hospital, 11 experienced persistent headaches for 5 days or more, before these resolved spontaneously, and two were readmitted to hospital (for post-lumbar puncture syndrome).

The number of patients included was too small, relative to the number of parameters investigated, for us to construct a model based on multivariate analysis to confirm the diagnostic relevance of the parameters described.

The various published scores and models were not tested in our study, as these were most often based on heterogeneous populations, particularly with regard to the age of the patients and the etiology of meningitis, sometimes retrospectively [8,10,36,43,44].

Although PCT appeared to be one of the most highly discriminative markers with regard to the differentiation of BM and VM, we did not study the impact of serum PCT level on the prescription or nonprescription of antibiotics. Antibiotic treatment was prescribed in the emergency unit to 31 patients in the VM group, even though all these patients had a serum PCT level < 0.12 ng/ml. In the pediatric context, a study focusing on the impact of serum PCT level on the duration of antibiotic treatment was performed during an epidemic of meningitis caused by enterovirus [45]. The treatment duration was found to be shorter by 2.4 days per patient in the group of patients with VM whose PCT level was determined 3 times per week.

Finally, we have not yet evaluated the impact of the results of real-time PCR testing for enterovirus on the prescription of antibiotics in patients with meningitis and a negative direct CSF examination.

## Conclusions

This study confirms the value of determining serum PCT and CSF lactate concentrations for the differential diagnosis of bacterial and viral meningitis, in the absence of microbiologic pointers at the time of the patient's emergency admission to hospital.

It would be interesting to study the impact of evaluating these two parameters on the prescription of antibiotics in patients admitted to the emergency unit with meningitis with clear CSF associated with a normal neurologic examination and a negative direct CSF examination.

## Key messages

• Identification of bacterial meningitis on direct examination had low sensitivity

• Identification of bacterial meningitis with classic biomarkers is insufficient

• Models for predicting the acute bacterial origin of meningitis are not easy to use

• Cerebrospinal fluid lactate and procalcitonin are easy to determine

• Cerebrospinal fluid lactate and procalcitonin are the best markers for differentiating between bacterial and viral meningitis

## Abbreviations

BM, bacterial meningitis; CRP, C-reactive protein; CSF, cerebrospinal fluid; PCR, polymerase chain reaction; PCT, procalcitonin; ROC, receiver operating characteristic; SD, standard deviation; VM, viral meningitis.

## Competing interests

The authors declare that they have no competing interests.

## Authors' contributions

AV, OM, and FZ conceived the study and designed the trial. AV, OM, and AB supervised the conduct of the trial and the data collection. MB, ND, JB, and BP undertook recruitment of patients and managed the data, including quality control. SG provided statistical analysis; FZ and JCB drafted the manuscript. All authors contributed substantially to its revision and approved the final manuscript.
